# Reading Assessment and Eye Movement Analysis in Bilateral Central Scotoma Due to Age-Related Macular Degeneration

**DOI:** 10.3390/jemr18050038

**Published:** 2025-08-30

**Authors:** Polona Zaletel Benda, Grega Jakus, Jaka Sodnik, Nadica Miljković, Ilija Tanasković, Smilja Stokanović, Andrej Meglič, Nataša Vidovič Valentinčič, Polona Jaki Mekjavić

**Affiliations:** 1 Eye Hospital, University Medical Centre Ljubljana, Grablovičeva 46, 1000 Ljubljana, Slovenia; andrej.meglic@kclj.si (A.M.); natasa.vidovic@kclj.si (N.V.V.); polona.jakimekjavic@kclj.si (P.J.M.); 2 Faculty of Medicine, University of Ljubljana, Vrazov Trg 2, 1000 Ljubljana, Slovenia; 3 Faculty of Electrical Engineering, University of Ljubljana, Tržaška c. 25, 1000 Ljubljana, Slovenia; grega.jakus@fe.uni-lj.si (G.J.); jaka.sodnik@fe.uni-lj.si (J.S.); nadica.miljkovic@etf.bg.ac.rs (N.M.); 4 School of Electrical Engineering, University of Belgrade, Bulevar Kralja Aleksandra 73, 11000 Belgrade, Serbia; ilijatanaskovic97@hotmail.com (I.T.); smiljastokanovic@gmail.com (S.S.); 5 The Institute for Artificial Intelligence Research and Development of Serbia, Fruškogorska 1, 21000 Novi Sad, Serbia; 6 Jozef Stefan Institute, Jamova Cesta 39, 1000 Ljubljana, Slovenia

**Keywords:** reading visual acuity, reading speed, near contrast sensitivity, microperimetry, preferential retinal locus, fixation stability, eye tracking, saccades, fixation duration

## Abstract

This study investigates reading performances and eye movements in individuals with eccentric fixation due to age-related macular degeneration (AMD). Overall, 17 individuals with bilateral AMD (7 males; mean age 77.47 ± 5.96 years) and 17 controls (10 males; mean age 72.18 ± 5.98 years) were assessed for reading visual acuity (VA), reading speed (Minnesota low vision reading chart in Slovene, MNREAD-SI), and near contrast sensitivity (Pelli-Robson). Microperimetry (NIDEK MP-3) was used to evaluate preferential retinal locus (PRL) location and fixation stability. Eye movements were recorded with Tobii Pro-glasses 2 and analyzed for reading duration, saccade amplitude, peak velocity, number of saccades, saccade duration, and fixation duration. Individuals with AMD exhibited significantly reduced reading indices (worse reading VA (*p* < 0.001), slower reading (*p* < 0.001), and lower near contrast sensitivity (*p* < 0.001)). Eye movement analysis revealed prolonged reading duration, longer fixation duration, and an increased number of saccades in individuals with AMD per paragraph. The number of saccades per paragraph was significantly correlated with all measured reading indices. These findings provide insights into reading adaptations in AMD. Simultaneously, the proposed approach in analyzing eye movements puts forward eye trackers as a prospective diagnostic tool in ophthalmology.

## 1. Introduction

Age-related macular degeneration (AMD) is a leading cause of vision impairment of elderly population over 60 years of age in the Western world, and the prevalence will continue to rise [1]. Consequently, AMD poses a significant public health problem with substantial socioeconomic implications due to the ageing population and increased longevity [2]. The disease progression and late-onset AMD lead to loss of central vision, resulting in permanent visual impairment. This has a profound impact on the patient’s quality of life and functional independence. Specifically, late-onset AMD with consequent central scotoma severely affects the patient’s ability to read [3]. In healthy vision, the fovea provides high acuity and serves as the locus for fixation, which is achieved through saccadic eye movements. Within six months, people with bilateral loss of foveal regions adopt an eccentric locus for fixation [4]. They use the extrafoveal regions of the retina to compensate for the loss of central fixation [5].

Eccentric fixation is an adaptation strategy where the image falls on the healthy paracentral areas of the retina based on the eye orientation. This non-central area of the retina is called the preferential retinal locus (PRL) [5,6]. To determine the PRL, the use of a system that simultaneously creates images of the retina and reproduces the stimulus is advised—microperimetry [5]. It has been demonstrated that reading performances are better when the PRL is located in the superior (inferior visual field) than in the inferior retina (superior visual field) [7]. The preference for the lower visual field is explained by the fact that we generally work with our hands in the lower visual field [7], the lower visual field is important in locomotion, and left-to-right readers need to monitor where their eyes have landed relative to the word previously fixated on the left [8].

Fixation stability in the PRL is compromised, as the density of cone photoreceptors falls off sharply as the distance from the foveola (eccentricity) increases, and convergence of the cone photoreceptors into a single ganglion cell also increases with eccentricity [9]. Previous studies have shown significant positive correlations between fixation stability and PRL distance from the former fovea [10,11], as well as a positive correlation between fixation stability and reading speed, demonstrating that fixation stability is desirable to improve reading performance [10,12].

During reading, the eyes traverse a line of text through a sequence of saccadic movements in the reading direction, interspersed with periods of fixations. Saccades are rapid, ballistic movements that bring an object of interest into the high-acuity fovea [4]. The fovea provides the origin of the oculomotor reference system for saccadic eye movements. Regular saccades follow a predictable relationship between amplitude, duration, and peak velocity, known as the main sequence. This pattern results from the brain optimizing eye movement planning [13]. When bilateral vision loss affects the fovea, it impairs visual acuity (VA) and disrupts eye movement control [4].

To date, various reports have attempted to explain saccadic eye movements within the context of shifting the oculomotor reference from the fovea to the PRL during reading [14,15,16] and visual search tasks [17,18,19]. However, the results are inconclusive. While some studies have shown slower saccades [16], others have reported an increased number of saccades [17,19]. On the contrary, Crossland and Rubin raised the alternative hypothesis that reading speed is a function of fixation duration and that changes in the number of saccades are artefactual [15]. Given this perspective, it is important to consider not only saccadic eye movements but also fixation behaviour when evaluating reading performance. Fixations represent the direction of gaze to a specific object for a period of time [20,21], and the duration of fixations is usually about 180–250 ms [22] for the healthy population. In contrast, longer and less stable fixations are expected in individuals with AMD [12].

This study examines reading indices (reading visual acuity (VA), reading speed, near contrast sensitivity) and eye movement characteristics (reading duration, saccade amplitude, peak saccade velocity, saccade duration, number of detected saccades per paragraph, and fixation duration) in individuals with bilateral central scotoma and an eccentric fixation due to late-onset AMD compared to controls with foveal fixation of similar age. We hypothesized that eye movement characteristics in individuals with bilateral central scotoma due to AMD would show strong correlation with reading indices. Additionally, we explored potential correlations between eye movement characteristics and functional (microperimetry) and structural (macular imaging) measures.

## 2. Materials and Methods

### 2.1. Patient Selection

We consecutively enrolled 17 individuals with bilateral late-onset AMD and an eccentric fixation (AMD Group) and 17 control individuals of similar age and a foveal fixation (Control Group). Individuals in the AMD Group were selected from those referred to the National Center for Comprehensive Rehabilitation of the Blind and Visually Impaired at the Department of Ophthalmology, University Medical Centre Ljubljana, Slovenia. Inclusion criteria for the AMD Group were: late-onset bilateral AMD—geographic atrophy or fibrotic scar in the fovea, a best-corrected visual acuity (BCVA) between 25 and 60 of an early treatment diabetic retinopathy study (ETDRS) of letters in the better-seeing eye, eccentric fixation assessed with microperimetry, and clear optic media. The Control Group were recruited from general ophthalmology outpatient clinic in Eye Hospital, University Medical Centre Ljubljana. The inclusion criterion for the Control Group was BCVA of >75 ETDRS letters on both eyes, because typically reading requires BCVA of at least 70 ETDRS for comfortable sustained reading. Exclusion criteria were other associated ocular pathologies that could affect the study outcome, such as macular lesion activity, optic neuropathy and moderate or severe cognitive decline. The study design and data analysis were based on the tenets of the Declaration of Helsinki. Written informed consent from all patients was obtained before testing. The study was approved by the National Medical Ethics Committee of the Republic of Slovenia on 20 June 2023 (No. 0120-246/2023/3).

### 2.2. Cognitive Impairment Screening

Given the potential impact of cognitive impairment on reading among elderlies, all subjects underwent a screening test to assess the scale of this effect. The Slovene version of Mini mental state examination (MMSE) was used, which is a 30-point questionnaire extensively used in clinical and research settings to measure cognitive impairment. Any score of 24 or more indicates normal cognition, and scores 19–23 indicate mild cognitive impairment. Below this, scores can indicate moderate (10–18 points) or severe (≤9 points) cognitive impairment [23], which were exclusion criteria in our study.

### 2.3. Reading Indices Assessment

ETDRS charts at a 2 m distance were used to measure BCVA at a distance monocularly, recorded as the logarithm of the minimum angle of resolution (logMAR). Reading indices (reading VA, reading speed, and near contrast sensitivity) were assessed in binocular conditions and compared with the binocular eye tracker testing. The Slovene version of the Minnesota low vision reading chart (MNREAD) charts (MNREAD-SI) at 40 cm interspace was used to obtain reading acuity (logMAR) and reading speed (words per minute) [24]. Near Pelli-Robson contrast sensitivity charts at 40 cm were used to measure near contrast sensitivity while reading, recorded as logarithm of contrast sensitivity (logCS). Participants were provided with Tobii Glasses Pro 2 correcting lenses adjusted for the testing distance of 40 cm. No additional low vision aids or specialized optical devices were used.

### 2.4. Microperimetry and Multimodal Macular Imaging

Fixation characteristics on PRL and PRL location were assessed using an MP-3 microperimeter (Nidek Technologies, NAVIS Software, Padua, Italy). Microperimetry was performed in all individuals in mydriasis via a 4-2 threshold strategy and a central 20-degree pattern with at least 37 stimuli presented on the fundus in about 200 ms against the white background with the luminance of 31.4 asb using an automated programme. The stimulus size was Goldmann III (a surface of 4 mm^2^). A single cross (red, max 1°) was used for the fixation target in the better-seeing eye in the AMD Group and both eyes in the Control Group. In the worse-seeing eye in the AMD Group, the fixation cross was corrected according to the VA. The contralateral eye was patched during the test. Fixation stability was acquired during an isolated fixation task (static fixation) in both eyes in the AMD and the Control Group, as well as additionally during microperimetry (dynamic fixation) in the better-seeing eye in the AMD Group [25].

According to the consensus established by Crossland and colleagues in 2011 [26], the PRL is defined as a limited area of functional retina that is repeatedly aligned with the visual target for a specific task, and which can also be used for attentional allocation and as an oculomotor reference. To determine the PRL, a system that simultaneously images the retina and presents the stimulus—microperimetry—is recommended [26]. The PRL was determined by continuous eye-tracking during fixation. The MP-3 mapped fixation points relative to the anatomical fovea and identified the retinal area most consistently used to maintain fixation. The centroid of this cluster of fixation points is designated as the PRL, and fixation stability is quantified by the dispersion of fixation points [5,26].

All subjects underwent optical coherence tomography (OCT) imaging and infrared and fundus autofluorescence (FAF) imaging by the Spectralis HRA-OCT Device (Heidelberg Engineering, Heidelberg, Germany).

Obtained fixation data were analyzed and compared using the Fujii classification [26] and the bivariate contour ellipse area (BCEA) quantification. By plotting the position of each fixation point on a Cartesian axis and calculating the area of an ellipse that encompasses a given percentage of fixation points, BCEA provides a precise continuous value with a smaller value, implying a more stable fixation [25]. The Fujii classification is a semi-quantitative method; the fixation stability is classified as being stable when 75% of fixation falls within a 2° circle, relatively unstable when 75% of fixation falls within a 4° circle, and unstable when less than 75% of fixation falls within a 4° circle [27].

**Figure 1:**
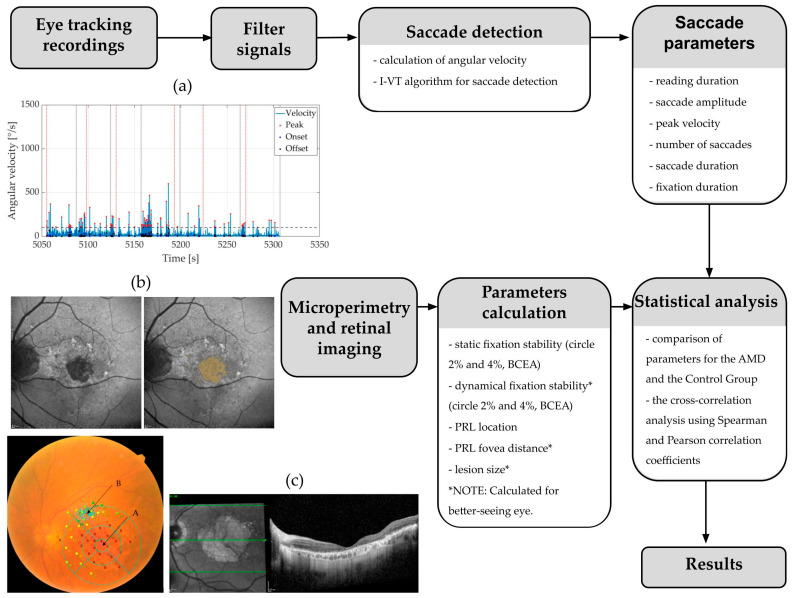
Diagram of the proposed methodology. (**a**) Detected saccades on angular velocity profiles. (**b**) FAF (**left**) and 50% DAF of the macula for lesion size measurement (**right**). (**c**) Microperimetry of the better-seeing eye with ETDRS grid for PRL location determination (**left**), OCT of the macula (**right**). FAF—fundus autofluorescence, DAF—decreased autofluorescence, I-VT—velocity threshold, BCEA—bivariate contour ellipse area, ETDRS—early treatment diabetic retinopathy study, OCT—optical coherence tomography, PRL—preferential retinal locus, AMD—age-related macular degeneration.

The ETDRS of a 6 × 6 mm grid was positioned at the centre of the fovea in the better-seeing eye of the AMD Group, according to OCT. The PRL was designated as the centroid of the cluster of fixation points determined during microperimetry. The distance from the fovea to PRL (PRL-fovea distance) was measured in mm. To determine the PRL location, the macula was divided into five fields using the ETDRS grid (including central, inferior, superior, nasal, and temporal fields) (Figure 1c).

From the 30° FAF images, areas of 50% decreased autofluorescence (DAF), representing fibrotic or atrophic macular lesions, were measured using our custom code written in MATLAB version 2024a (The MathWorks, Inc., Natick, MA, USA). According to the ProgStar criteria [27], 50% DAF lesions were defined as being at least 50% as dark as the optic nerve head or main blood vessels, which serves as the reference for 100% darkness on the grayscale. The opposite reference point represented a healthy retina (0%). Each lesion had a minimum diameter of 125 µm, which is approximately the width of a major retinal vein. The lesion was visibly darker than the surrounding background autofluorescence [28]. The area of 50% DAF was measured in mm^2^ (Figure 1b).

### 2.5. Eye Tracking Recordings and Eye Movement Analysis

A head-mounted mobile eye-tracker system, the Tobii Pro-glasses 2 (Tobii Llc., Stockholm, Sweden) [29], was used to track eye movements at horizontal and vertical visual angles. The Tobii eye tracker operated at a sampling rate of 50 Hz. Concurrent audio and video recordings were collected to precisely identify reading intervals, while raw eye-tracking data were collected and stored for further analysis. Tobii Pro-glasses 2 [29] were calibrated individually for each participant and additionally equipped with correcting lenses to collect accurate eye-tracking data. During the calibration process, the subject remained focused on the centre of the calibration target. For individuals in the binocular vision in the AMD and Control Group, eye tracking data were recorded while reading the MNREAD test. The eye movement analysis was separately performed for reading strophes on a two-sided board and separately performed for reading the first three strophes for participants from both the AMD group and the Control Group.

Sampling frequency significantly influences the accuracy of eye movement detection [30,31], particularly for fast, transient events such as saccades. High-frequency systems (e.g., ≥250 Hz) are generally recommended to ensure precise measurement of saccade onset, offset, peak velocity, and duration. Despite these limitations, research has shown that certain metrics-such as mean fixation duration and general saccade counts-can still be reliably estimated at 50–60 Hz, especially with the aid of preprocessing methods like interpolation or noise filtering [32,33,34].

The eye tracker data comprised timestamps and the horizontal, as well as the vertical coordinates of participants’ gaze locations. The recorded data consisted of coordinates that represent the proportion of paper width and height at which the participants’ gaze is directed. Prior to analysis, the recorded eye tracking data undergoes preprocessing to eliminate noise and prepare the data for further analysis using Python version 3.9 [35]. To ensure equidistant sampling, impulse noise corresponding to the abrupt changes exceeding 20° between consecutive samples, which was physiologically implausible, was eliminated from the recording. Noise originates from optical artefacts (e.g., eyelash occlusion or momentary loss/misdetection of the pupil or corneal reflection) rather than from eye movements [36]. A 20° threshold is chosen such that at a 50 Hz sampling rate, such a change in amplitude implies an angular speed of 1000°/s, which is faster than what is physiologically possible and likely due to artefacts, non-biological in origin as per Holmqvist et al. [36]. Linear interpolation was conducted at the boundaries of the affected segment, effectively replacing impulse noise with interpolated samples. The start and the end of each strophe (i.e., paragraph) on the first and the second page of the reading board were manually determined from the timestamps within the audio-video recordings. Data were acquired in a normalized form (ranging from 0 to 1) for the appropriate calculation of visual angles, the mean was removed, and then the data were scaled using factors of 82 for the horizontal axis and 52 for the vertical axis, which represent the maximal fields of view in degrees for the Tobii Pro-glasses 2 [29]. The eye tracker data, with impulse noise removed and converted into visual angles, were then preprocessed for proper detection of saccades and fixations in MATLAB. Signals were filtered using a median filter with a window length of 60 ms to reduce high-frequency noise [33]. Then, horizontal and vertical angular velocities were calculated using a central derivative based on visual angles from the eye tracker [37]. The eye movement analysis was performed using both horizontal and vertical gaze coordinates recorded by the Tobii eye tracker. The analysis was conducted on the magnitude of angular velocity $\dot{\theta }$, which is calculated as shown in Equation (1):

(1) $$\dot{\theta }=\sqrt{{\left(\dot{x}\right)}^{2}+{\left(\dot{y}\right)}^{2}}$$

where $\dot{x}$ and $\dot{y}$ represent the horizontal and vertical angular velocities, respectively. In addition to the global velocity-based analysis, we also performed separate analyses for horizontal and vertical eye movements. Saccades were identified using the so-called velocity threshold (I-VT) approach, which is a class of simple, yet highly effective algorithms for saccade detection and eye movement data analysis [38]. Specifically, we used the adaptive Nyström-Holmqvist (NH) algorithm [37] with it being a well-established I-VT method [39]. The NH algorithm was used to delineate three characteristic points from the total angular velocity, required for the subsequent calculation of the eye movement parameters: peak saccadic velocity, the onset of the saccade, and the offset of the saccade. Firstly, the adaptive threshold was iteratively determined based on the distribution of eye tracker data [37]. The initial threshold was set to 200°/s. If the threshold fell below 100°/s, the algorithm was considered non-convergent and the threshold reverted to 100°/s. The localization of saccade peaks is conducted using the MATLAB findpeaks function to determine local extrema, with a minimum inter-saccadic interval of 50 ms [40] and a peak amplitude above the final threshold calculated using the proposed method [37]. The process repeated until the difference between consecutive threshold estimates was less than 1°/s [37]. For saccade onsets, the algorithm tracked backward from each velocity peak to the earliest point exceeding the onset threshold and then located the nearest local minimum as the onset. A similar approach was used for the detection of saccadic offsets, though the threshold was slightly differently defined [37].

Delineated saccadic events were used for the calculation of 5 features for further statistical analysis: saccade amplitude, peak saccade velocity, saccade duration, number of detected saccades per paragraph, and fixation duration. The reading duration for each strophe was assessed by calculating the time difference between manually identified start and end timestamps in the Tobii video recordings. For each subject, the mean, standard deviation (SD), and median of the parameter of all saccades were calculated separately for reading both pages and for the first three paragraphs, and these values were used in the subsequent statistical analysis. All parameters that were calculated in this study are presented in Table 1.

### 2.6. Statistical Analysis

All steps of statistical analysis were conducted using the Python 3.9 programming language [35]. During preprocessing, two missing (Not Available—NA) values were detected (ID64: fixation duration; ID24: ETDRS for the worse eye) and imputed using the median value of their respective diagnostic group (AMD or Control Group). The parameters were categorized into three groups, as shown in Table 2, each requiring distinct statistical approaches due to their inherent characteristics.

The first group comprised measures that are not eye-specific and were compared directly between the AMD and Control Group. Descriptive statistics (mean ± SD and median with range [min, max]) were calculated separately for both groups. Normality of the parameters was evaluated using the Shapiro–Wilk test. Depending on the distribution, independent-samples t-tests (parametric) or Mann–Whitney U tests (nonparametric) were applied. Resulting *p*-values underwent Benjamini–Hochberg false discovery rate (FDR) correction for multiple comparisons, with effect sizes reported as Cohen’s *d* (parametric) or Cliff’s *δ* (nonparametric).

The second group contained eye-specific parameters, yielding four subgroups: control left eye, control right eye, AMD better eye, and AMD worse eye. We compared the two control eyes using the same workflow as for non-eye-specific parameters, finding no significant difference. Due to the results, we selected the right eye to represent the Control Group. Subsequent analyses compared parameters for right eyes in the Control Group, as well as the better eyes and the worse eyes in the AMD Group. After assessing normality via Shapiro–Wilk, one-way Analysis of Variance (ANOVA) (parametric) or Kruskal–Wallis tests (nonparametric) were employed, followed by Tukey’s Honestly Significant Difference (HSD) or Dunn’s post hoc tests with Benjamini–Hochberg FDR adjustment. Pairwise effect sizes (Cohen’s *d* or Cliff’s *δ*) were reported for all comparisons.

The third group included parameters that exist only for the AMD Group and, therefore, could be compared against the Control Group. These parameters were summarized using descriptive statistics (mean ± SD and median with range [min, max]) for the AMD Group alone.

The Shapiro–Wilk test was used to check the normality of datasets [45]. Since data did not conform to the normality assumption, we applied the nonparametric Aligned Rank Transform (ART) procedure for multiple comparison and for evaluating possible interactions. ART was applied together with the ART contrasts as a post hoc test with two groups of individuals (the AMD and Control Group), as well as for reading different paragraphs (the first three paragraphs) as factors (both groups of subjects and the paragraphs are set as factors) [46,47]. For all tests, the *p*-value significance level was set to 0.05. Only the first three paragraphs were compared. Also, this particular analysis (per paragraph) did not include imputation of missing values. For post hoc *p*-values, Tukey adjustment was applied.

Statistical analysis was conducted on horizontal and vertical eye-tracker features independently between the AMD and Control groups using median values, with group comparisons performed using either Welch’s t-test or the Mann–Whitney U test, depending on the distribution assessed by the Shapiro–Wilk test. These features were extracted from the eye-tracker signal during the reading of the first page.

For all statistically significant outcomes for initial differentiation between the Control Group and AMD, a One-way Analysis of Covariance (ANCOVA) test was used with age as a covariate after homogeneity of regression slopes. Multiplicity was controlled using the Benjamini–Hochberg false discovery rate (FDR) procedure, while effect sizes were reported as partial *η*^2^ [48,49,50]. Pairwise associations were evaluated using both Pearson and Spearman correlation coefficients. Two separate analyses were conducted. The first focused on the AMD group and examined cross-correlations among features including PRL–fovea distance, AMD lesion size, and both static and dynamic fixation parameters. Notably, dynamic fixation measures were available exclusively for participants in the AMD group, requiring a group-specific analysis. The second analysis assessed cross-correlations across the entire cohort, including both the AMD and Control groups. This analysis was not eye-specific and included reading indices along with two eye tracker features: average fixation duration and number of saccades per paragraph. Results were visualized as a heatmap, where colour gradients represent correlation coefficients (rounded to two decimal places), and symbols denote significance levels: * *p*-value ≤ 0.05, ** *p*-value ≤ 0.01, *** *p*-value ≤ 0.001, and ‘ *p*-value ≤ 0.1.

## 3. Results

The mean age of the AMD Group was 77.47 ± 5.96 years (range 64–84 years) and 72.18 ± 5.98 years (range 63–81 years) in the Control Group. The difference in age was statistically significant (*p* = 0.03). There was female predominance in the AMD Group (7 males) and male predominance in the Control Group (10 males). In the AMD Group, the mean BCVA at distance in the better-seeing eye was 0.79 logMAR ± 0.21 SD (range 0.50–1.20 logMAR) and in the worse-seeing eye 1.30 logMAR ± 0.40 SD (range 0.80–2.30 logMAR). In the Control Group, the mean BCVA at distance was on the right eye 0.07 logMAR ± 0.09 SD (range 0.00–0.20 logMAR) and on the left eye 0.06 logMAR ± 0.08 SD (range 0.00–0.20 logMAR).

### 3.1. Reading Indices (Reading VA, Reading Speed, and near Contrast Sensitivity)

As shown in Table 3, all the reading indices showed significant differences between the AMD and the Control group. The AMD Group had a mean reading VA of 0.95 ± 0.22 logMAR, compared to 0.18 ± 0.20 logMAR in the Control Group. Reading speed was also reduced in the AMD Group, averaging 37.12 ± 21.95 wpm, while the Control Group averaged 142.65 ± 42.79 wpm. Near contrast sensitivity was lower in the AMD group (1.03 ± 0.27 logCS) than in Control Group (1.65 ± 0.14 logCS). All differences were statistically significant (*p* < 0.001). All effects that were statistically significant in the initial analysis remained significant after adjusting for age and applying FDR correction. All reading indices (reading visual acuity, reading speed, and near contrast sensitivity) showed an adjusted *p*-value of <0.001 with partial *η*^2^ > 0.60, which is considered a large effect size.

Table 4 presents the results for eye-specific parameters comparing the right eye of the Control Group with the better-seeing and worse-seeing eyes of the AMD Group. As all variables exhibited non-normal distributions based on the Shapiro–Wilk test, the Kruskal–Wallis test was applied, revealing statistically significant differences across all parameters (*p*-value < 0.001). Dunn’s post hoc analysis indicated significant pairwise differences for all comparisons, except for those between the better-seeing and worse-seeing eyes in the AMD Group for BCEA 1 sd to 3 sd, which showed borderline significance.

### 3.2. Fixation Stability

The descriptive statistics of static (Table 3) and dynamic fixation parameters (Table 5) were extracted from the better-seeing eye in the AMD group. All static fixation stability data was statistically significantly different between the AMD and the Control Group (comparing the better-seeing eye in the AMD Group and right eye in the Control Group) according to the Fujii classification and BCEA values using MP-3 perimeter (*p* < 0.05) (Table 3). There was no statistically significant difference in static fixation stability data between the right and left eye in the Control Group (*p* > 0.72). When comparing static fixation parameters between the better-seeing and the worse-seeing eye in the AMD Group, all *p*-values showed significant differences (*p* < 0.05). Dynamic fixation stability was markedly reduced compared to static fixation in the AMD group (Table 5). In dynamic conditions, the mean percentage of fixation points within the central 2° was 34.76%, and within 4° was 65.12%, both substantially lower than the corresponding static fixation values (66.47% and 89.71%, respectively). BCEA values also reflected significantly greater instability under dynamic conditions, with mean areas of 17.82°^2^, 47.96°^2^, and 91.79°^2^ for 1, 2, and 3 standard deviations, respectively. These were considerably higher than the BCEA values observed during static fixation (4.45°^2^, 11.98°^2^, and 22.92°^2^, respectively).

### 3.3. PRL Characteristics (Location, Distance from the Fovea) and AMD Lesion Size

Eleven out of 17 individuals (64.70%) in the AMD Group showed the PRL in the superior field of the macula in the better-seeing eye (Figure 1b). PRL location in other fields of the macula was as follows: three subjects (17.65%) in the nasal field, two subjects (11.76%) in the central field, and one subject (5.88%) in the temporal field of the macula. In 11 AMD individuals (64.70%), the PRL was located in the same macular field in both eyes. Among these, 10 individuals (90.90%) had PRLs located in the superior macular fields bilaterally. One individual demonstrated PRLs in the temporal macular fields of both eyes. The mean PRL-fovea distance in the better-seeing eye of the AMD Group was 1.69 ± 0.99 mm. In the Control Group, all subjects had foveal fixation in both eyes. The area of the AMD lesion size (fibrotic or atrophic macular lesion) in the better-seeing eye in the AMD Group was 12.34 ± 6.07 mm^2^ (range 2.25 mm^2^–21.17 mm^2^) (Table 5).

### 3.4. Eye Movement Analysis

**Figure 2:**
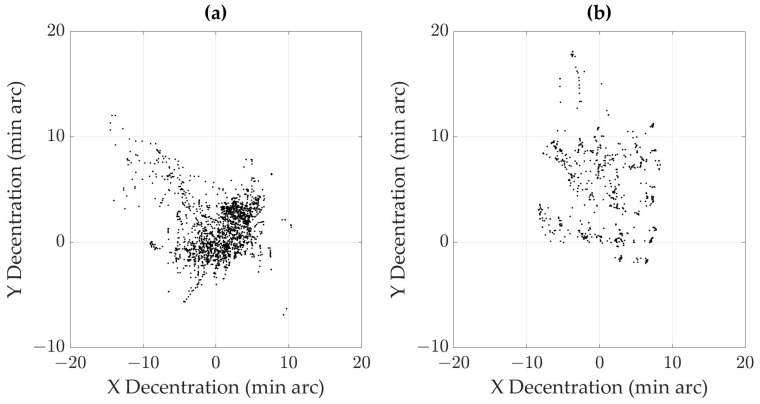
Scatter plots of horizontal and vertical gaze points during reading the first three paragraphs. Each point on both plots corresponds to an individual gaze sample, with the abscissa representing horizontal gaze displacement (X Decentration) and the ordinate representing vertical gaze displacement (Y Decentration) relative to the screen centre and expressed in min arc units (1/60 of a degree). The data are presented for a representative participant from the AMD Group (**a**) and for a sample subject from the Control Group (**b**).

The participants from the AMD Group exhibit variable gaze stability, particularly in the horizontal direction. This is probably due to more frequent eye movements across the text compared to the gaze points of the participants from the Control Group. Obtained gaze distributions suggest that the participants from the AMD Group have less stable and more variable fixations, and scan more frequently during reading. To illustrate this, we present the horizontal and vertical positions of gaze points recorded on a sample participant from each group while reading the first three paragraphs in Figure 2.

The differences between the AMD and Control Group influenced reading duration (*p* < 0.001), while the paragraph in the MNREAD test (*p* = 0.210) and the interaction between paragraph and subject group (*p* = 0.329) did not influence the reading duration. Contrast tests confirmed that only differences among the AMD and the Control Group are present (all *p*-values are <0.0001), while there were no differences among the AMD and Control Group for reading different paragraphs in the MNREAD test.

No significant differences were indicated between the AMD and Control Group or among paragraphs for saccade amplitude, peak saccade velocity, and saccade duration. These findings are consistent with results obtained when horizontal and vertical movements were analyzed together. Additionally, no significant interactions between paragraph and subject group were detected for these measures.

A significant difference in fixation duration was found between the AMD and Control Group (*p* < 0.001), while paragraph content (*p* = 0.137) and the interaction between paragraph and subject group (*p* = 0.105) did not significantly affect fixation durations.

Significant differences were observed in the number of detected saccades per paragraph between groups (*p* < 0.001), with very low differences among paragraphs (*p* = 0.090) and some interaction between a paragraph and subject group factors (*p* = 0.036). Specifically, the AMD Group reading the first paragraph exhibited a statistically different number of saccades than the Control Group reading paragraph 3 (*p* = 0.027). Other comparisons between the AMD and Control Group across paragraphs 1 and 2 did not reach statistical significance (*p*-values = 0.062 and 0.140, respectively). The significant difference in the number of saccades per paragraph is confirmed after adjustment for age as a covariate with adjusted *p*-value = 0.022 and partial *η*^2^ = 0.16, indicating a large effect size.

**Figure 3:**
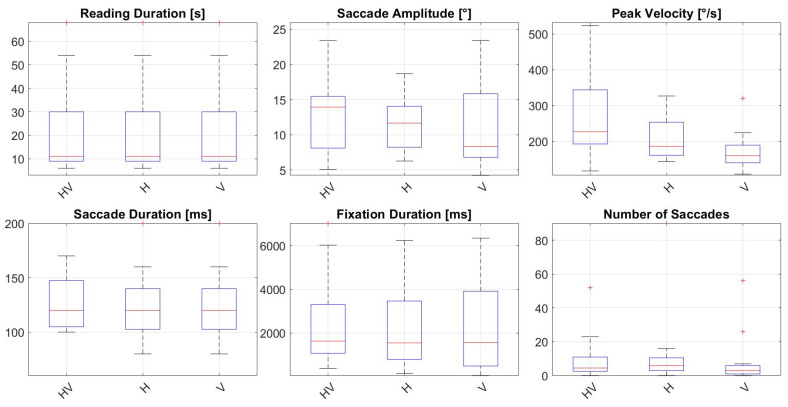
Eye tracker parameters for the AMD Group during reading of the first paragraph. HV—horizontal and vertical, H—horizontal, V—vertical, s—seconds, ms—milliseconds, ° degrees of visual angle, °/s—degrees per second.

**Figure 4:**
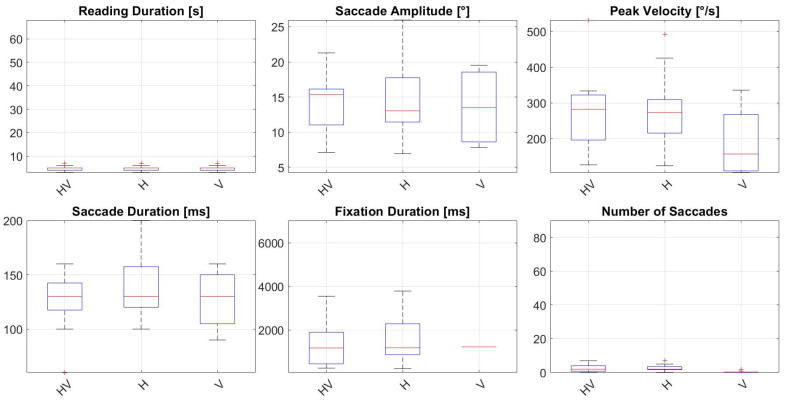
Eye tracker parameters for the Control Group during reading of the first paragraph. HV—horizontal and vertical, H—horizontal, V—vertical, s—seconds, ms—milliseconds, ° degrees of visual angle, °/s—degrees per second.

Additional analysis of horizontal and vertical saccadic eye movements revealed significantly more horizontal and vertical saccades, as well as longer fixation durations, in the AMD group compared to the Control Group. Fixation durations in the AMD group were 2145.33 ± 1867.84 ms (horizontal velocity) and 2283.75 ± 2327.34 ms (vertical velocity), versus 1516.43 ± 1014.27 ms and 1120.00 ± 0.00 ms in the Control Group, respectively. The number of saccades was also higher in the AMD group (horizontal: 11.75 ± 21.34; vertical: 7.41 ± 13.91) than in the Control Group (horizontal: 2.71 ± 1.90; vertical: 0.35 ± 0.70). Horizontal saccade amplitudes were smaller in the AMD Group (11.58 ± 3.58°) compared to the Control group (14.07 ± 4.90°). Results of the eye tracker parameters for the AMD Group are showed in Figure 3 and for the Control Group in Figure 4.

### 3.5. Correlation Between Eye Movements, Reading Indices, Microperimetry, and Macular Imaging Findings

The reading duration showed a significant positive correlation with the AMD lesion size in the better-seeing eye of the AMD group, as confirmed by both Spearman’s rank correlation (*ρ* = 0.55, *p* ≤ 0.05) and Pearson’s correlation coefficient (*r* = 0.52, *p* ≤ 0.05).

**Figure A1:**
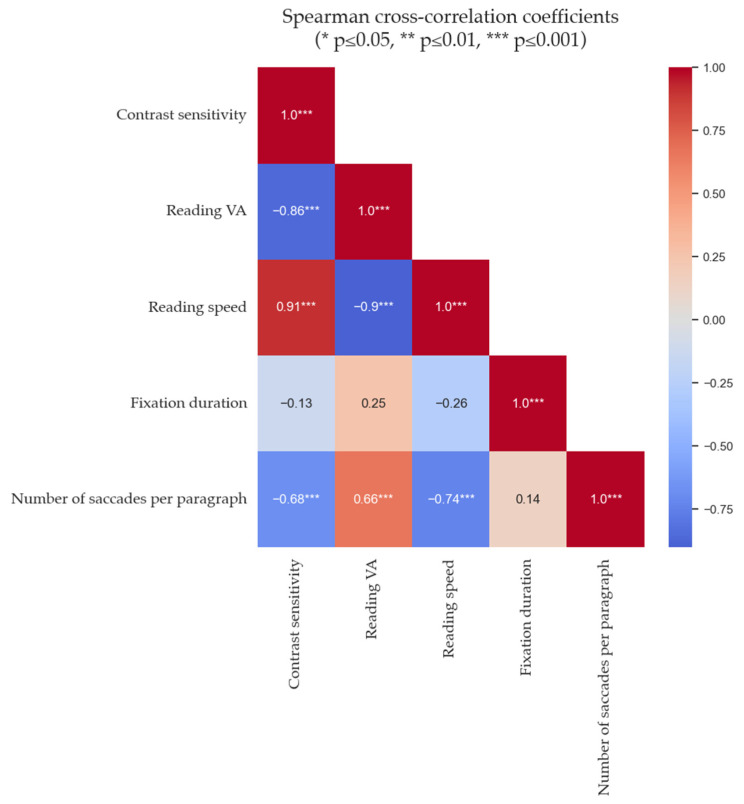
Spearman cross-correlation coefficients in the AMD and Control Groups.

**Figure A2:**
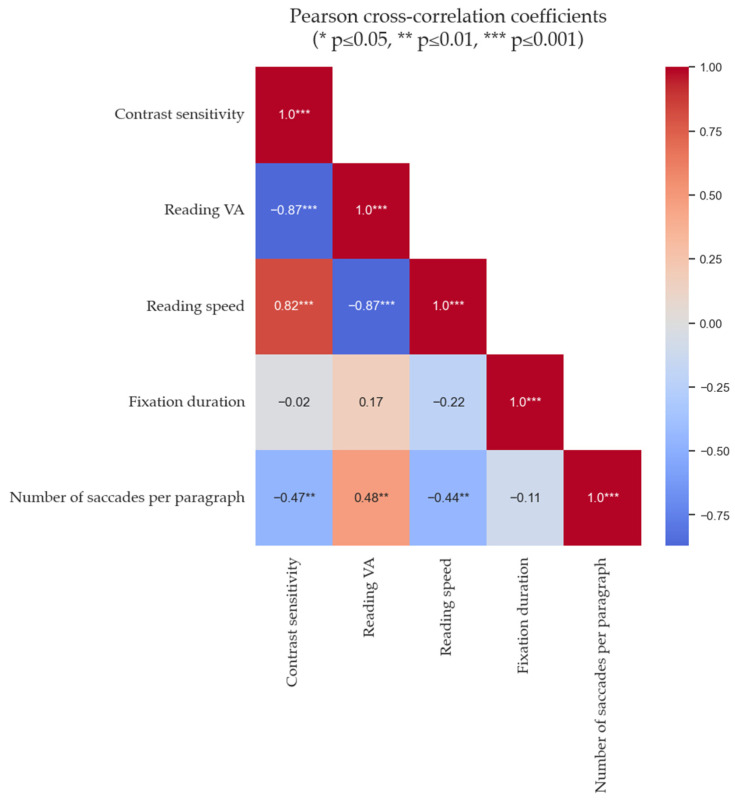
Pearson cross-correlation coefficients in the AMD and Control Groups.

The fixation duration showed no significant correlation with reading indices, as indicated by both Spearman’s rank correlation and Pearson’s correlation analyses including the AMD and Control Group (all *p* > 0.05) (Figure A1 and Figure A2).

The number of detected saccades per paragraph demonstrated significant positive correlations with all measured reading indices including the AMD and Control Group. According to Spearman’s rank correlation, the number of saccades per paragraph was strongly correlated with reading VA (*ρ* = 0.66, *p*-value ≤ 0.001), reading speed (*ρ* = 0.74, *p*-value ≤ 0.001), and near contrast sensitivity (*ρ* = 0.68, *p*-value ≤ 0.001). Pearson’s correlation analysis also confirmed moderate positive associations, with correlation coefficients of *r* = 0.48 (*p*-value ≤ 0.01) for reading VA, *r* = 0.44 (*p*-value ≤ 0.01) for reading speed, and *r* = 0.47 (*p*-value ≤ 0.01) for near contrast sensitivity (Figure A1 and Figure A2).

**Figure A3:**
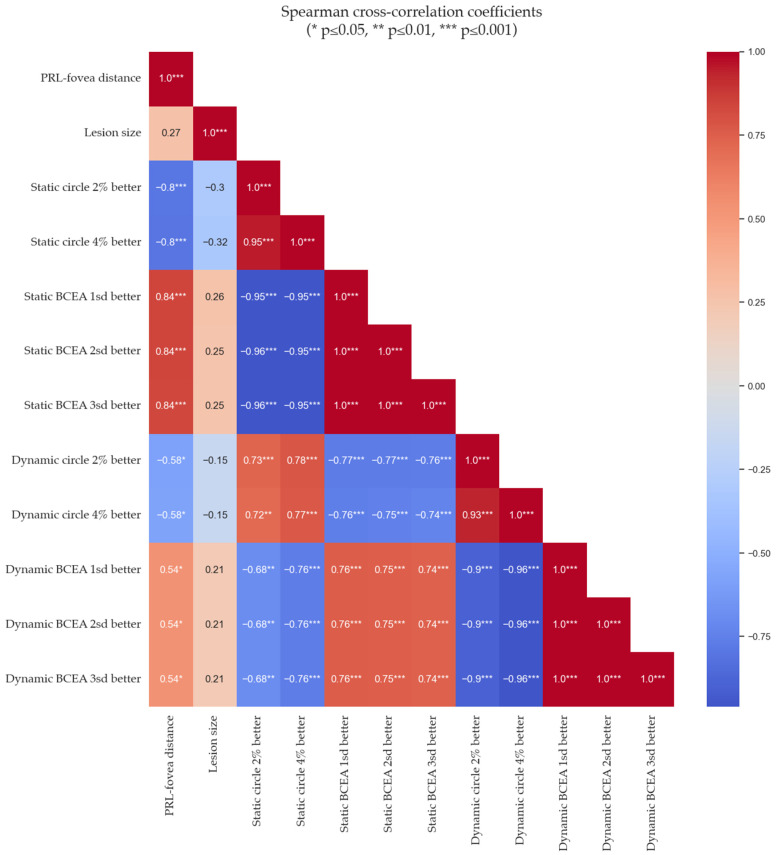
Spearman cross-correlation coefficients in the AMD Group.

**Figure A4:**
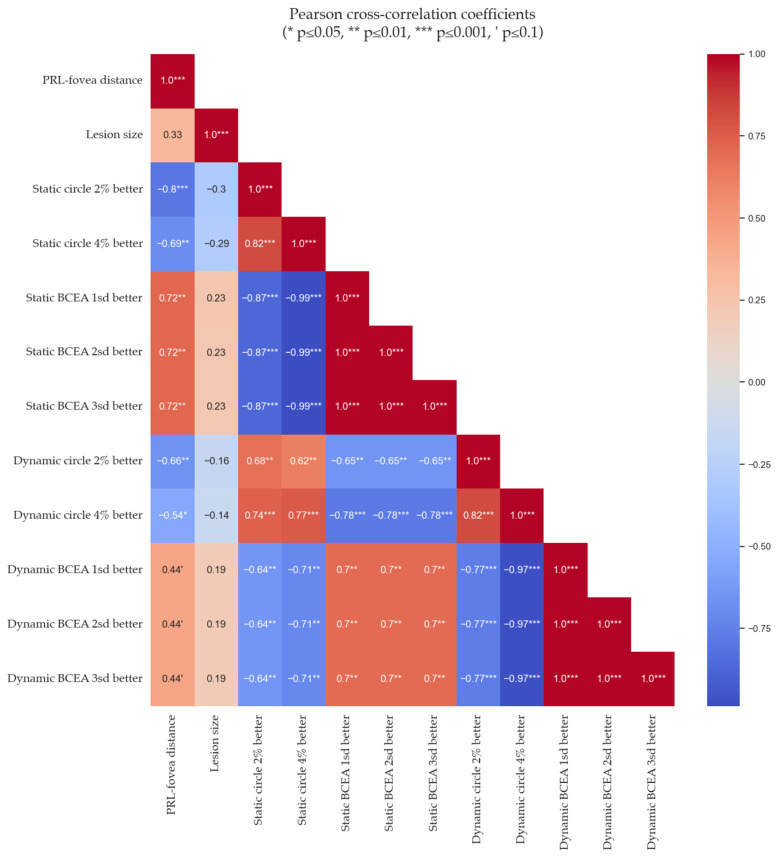
Pearson cross-correlation coefficients in the AMD Group.

There was a strong negative correlation between the PRL-fovea distance in the better-seeing eye of the AMD Group and static fixation. A strong association was observed with the Fujii classification (Spearman’s ρ = 0.80, *p*-value ≤ 0.001; Pearson’s r = 0.80 and 0.69, *p*-value ≤ 0.01) as well as with BCEA, where Spearman’s ρ = 0.84 (*p*-value ≤ 0.01) and Pearson’s r = 0.72 (*p*-value ≤ 0.001). PRL–fovea distance showed a moderate but statistically significant correlation with dynamic fixation stability (Spearman’s ρ = 0.58 for the Fujii classification and ρ = 0.54 for BCEA; Pearson’s *r* = 0.54–0.66 for the Fujii classification and *r* = 0.44 for BCEA), with *p* ≤ 0.05 for all parameters except Pearson’s correlation with BCEA (Figure A3 and Figure A4).

## 4. Discussion

Our study confirmed that individuals with AMD exhibited significantly poorer reading VA, slower reading speed, and reduced near-contrast sensitivity compared to control subjects. Eye movement analysis further revealed prolonged reading duration, longer fixation duration, and an increased number of saccades per paragraph in the AMD Group. Notably, the number of saccades per paragraph was significantly correlated with all measured reading indices.

The increased number of saccades observed in our AMD Group aligns with prior studies documenting atypical eye movement patterns in individuals with central vision loss. While most previous research has investigated eye movement behaviour during visual search tasks [17,18,19], our study focuses on reading—a fundamental skill essential for maintaining independence and quality of life. Investigating reading under real-life conditions in our study offers valuable insights into the specific challenges faced by individuals with AMD, facilitating the development of targeted rehabilitation strategies to enhance reading performance. Our study, which was based on a reading task, found an increased number of saccades alike other studies based on during visual search tasks. For instance, Vullings et al. found that individuals with binocular scotoma made more saccades toward their scotoma and showed frequent backwards saccades during visual search tasks [19]. Similarly, Shandize et al. reported more variable saccade directions and an increased number of saccades in non-target directions during smooth pursuit, which increased with PRL eccentricity [17]. Van der Stigchel et al. observed that individuals with AMD had increased search latencies associated with an increased number of saccades, decreased saccadic amplitudes, and longer saccadic intervals [18]. Recent research by Yu and Kwon revealed that the proportion of regressive saccades increased linearly with the size of the central scotoma. Comparing the number of regressive saccades between the Control Group and AMD Group presents a promising avenue for future research [51].

In a reading context, additional evidence for altered eye movements was provided by Giacomelli et al., showing that individuals with bilateral scotoma due to Stargardt disease exhibited significantly slower saccadic movements compared to controls, and that saccade speed tended to correlate positively with reading performance [16]. However, Crossland and Rubin suggested that the increased number of saccades may not be a direct cause of difficulty in reading, but rather a consequence of slower reading itself [15]. This perspective raised the possibility that reading speed is primarily driven by fixation duration, and that variations in a number of saccades might be incidental rather than causal. Therefore, a comprehensive evaluation of reading performance should include saccadic eye movements and fixation behaviour [15].

In our study, individuals with AMD showed significantly prolonged fixation durations as opposed to the findings of Crossland and Rubin [15], who reported no significant difference in fixation duration between patients and controls. Additionally, changes in fixation duration were not related to changes in reading speed [15]. On the other hand, Calabrese et al. identified that fixation duration had a significant effect on reading speed and fixation duration was negatively correlated to reading speed [14]. However, our results showed no correlation between fixation duration and reading speed in AMD Group.

Higher numbers of both horizontal and vertical detected saccades in our AMD Group indicate a less structured and more multidirectional scanning strategy, likely reflecting compensatory mechanisms in response to central vision loss. The observed oculomotor patterns align with the notion that individuals with AMD adopt atypical reading strategies that diverge from the conventional left-to-right sequence. As noted by Harvey and Walker [52], the presence of a central scotoma disrupts this standard reading pattern, often resulting in irregular eye movements that include backward, forward, and vertical shifts. This altered strategy supports the interpretation that many of the fixations recorded in the AMD Group did not land directly on the intended text, but instead reflect an adaptive response aimed at locating and processing words using peripheral vision.

Horizontal saccade amplitudes were smaller in our AMD Group compared to the Control Group. This finding is consistent with the perceptual span hypothesis in AMD, which proposes that central vision loss reduces and distorts the span of visual information accessible during fixation, leading to inefficient reading strategies and increased reliance on compensatory eye movements [14,15].

Previous studies have demonstrated that poorer fixation stability is closely associated with slower reading speed in individuals with central vision loss [10,12]. Altinbay et al. and Tarita-Nistor et al. highlighted that the PRL-fovea distance plays a crucial role in fixation stability. Specifically, a greater PRL–fovea distance was associated with more unstable fixation [10,11]. This finding is consistent with our results, which showed that greater distances from the fovea were associated with reduced fixation stability.

In our group of AMD individuals, the PRL was predominantly located in the superior field of the macula in both eyes, corresponding to the inferior visual field. This finding is consistent with previous reports suggesting that a superior PRL location is more advantageous for reading, likely due to better alignment with reading direction and oculomotor strategies [7]. However, the proportion of individuals with a superior PRL in our study was notably higher (65%) than reported in earlier studies—49% in Farzaneh et al. [53], 41% in Tarita-Nistor et al. [11] and 26% in Altinbay et al. [10]. This could be because all our AMD participants had long-standing macular lesions in both eyes (documented for more than a year), allowing sufficient time for adaptation and the establishment of a PRL in a more functionally optimal location.

When evaluating reading performances in individuals with AMD, our results indicate that the AMD lesion size is more closely related to reading duration than standard reading indices such as reading VA, reading speed, and near contrast sensitivity. Our results suggest that larger macular lesions may increase the time required to process text. Since reading VA and reading speed may not fully capture the functional burden of reading with central vision loss, reading duration appears to be an important indicator of reading performance in individuals with AMD.

Unlike most previous studies that analyzed eye movements during visual search [17,18,19], our study evaluated eye movements specifically during reading in a real-world setting. In contrast to studies that investigated eye movements in individuals with both AMD and Stargardt disease [14,15,16], our study focused on a homogeneous group of individuals with long-standing AMD (over one year), allowing for the observation of stable adaptive reading strategies. Importantly, the proposed multimodal approach—combining assessment of reading indices, microperimetry, structural macular imaging, and eye tracking —provides valuable insight into how AMD lesion size, PRL location, and PRL–fovea distance influence oculomotor behaviour and reading performance.

The results of our study can contribute to the further development of effective strategies for vision rehabilitation. These include microperimetric biofeedback training to optimize PRL location and to improve fixation stability on PRL [54]. Eye tracking could be integrated into current clinical practice in low vision clinics as a promising diagnostic tool for a complex assessment of visual functioning. In addition to reading ability, eye-tracking could also be used to assess walking orientation, ability to work at a computer and in activities of daily living in a real-world setting for various causes of visual impairement. From a clinical standpoint, eye-tracking offers a unique opportunity to move beyond traditional measures like visual acuity and contrast sensitivity by providing objective, quantifiable insights into how patients with AMD and other low vision conditions function visually. This can support more individualized rehabilitation planning. Although eye tracking presents a promising tool, it should be used with caution in clinical and medical contexts due to potential variability in data quality, calibration issues, and participant-related factors that may affect the reliability of the measurements [55]. Data processing and interpretation currently require significant time, technical expertise, and are still relatively costly. Future developments in automated processing and integration with clinical imaging could pave the way for broader implementation of eye tracking in ophthalmologic diagnostics and monitoring of vision rehabilitation outcomes.

A major limitation of this study design refers to the use of Tobii eye tracking system. Despite its popular usage in recent decades, the mobile eye trackers demonstrated low data quality [56]. Previous studies identified several limitations associated with the use of the Tobii eye tracker: (1) data loss (high data loss can occur due to various issues such as tilted glasses, pauses in recording or frozen gaze [57]), (2) slippage (head movements can cause slippage, affecting the accuracy of gaze tracking [58]), (3) gaze accuracy (the accuracy of gaze direction is altered [58]), (4) sampling frequency (sampling frequency of 50 Hz limits temporal resolution, which may hinder precise fixation and gaze detection [31,37,56]), and (5) scene instability (variations in distances and scene instability can affect the quality of the recorded signals [59]). In individuals with bilateral central scotoma achieving reliable calibration of the eye tracking system can be challenging due to eccentric fixation and unstable fixation. The calibration process involved the patient fixating on a target while the investigator optimized pupil positioning by adjusting the nose pads and monitoring the raw video stream from the infrared cameras in the glasses. All enrolled individuals successfully completed the calibration process, ensuring the quality of eye-tracking data.

Further limitation is that microperimetry was not performed during the reading task itself. Consequently, it was assumed that the PRL identified by microperimetry corresponded to the PRL used during reading. There is also a concern regarding the method used to quantify AMD lesion size. The AMD lesion area was calculated using custom MATLAB software that applied the ProgStar criteria to FAF images. While this method provides objective and reproducible measurements, it may be less accurate for delineating fibrotic lesions, which often exhibit heterogeneous FAF characteristics. As a result, some variability in lesion area estimation, particularly for fibrotic components, must be acknowledged. Another limitation is in the statistical analysis of number of detected saccades per paragraph, where statistical significance was observed between paragraph 1 in the AMD Group and paragraph 3 in the Control Group. Although the comparison between paragraph 1 (AMD group) and paragraph 3 (Control group) does not follow a typical within-paragraph, between-group structure, it reflects the strongest observed contrast in saccade behaviour across all group and paragraph combinations. Importantly, the paragraphs were matched for length, complexity, and layout, making cross-paragraph comparisons interpretable. We acknowledge that ideally group comparisons would be made within the same paragraph. However, this specific contrast (AMD-paragraph 1 vs. Control-paragraph 3) emerged as the statistically significant pairwise difference after correction. Given that paragraph content was carefully controlled, this comparison highlights the sensitivity of saccade metrics to group-level visual status and warrants reporting.

Acknowledging sex differences as a potential factor in AMD is a limitation of the present study, as our sample size limits meaningful stratification. However, given the conflicting evidence on gender-related AMD prevalence and the possible roles of both biological and socioeconomic factors, this remains an important direction for future research on larger, sex-balanced cohorts [60,61]. Finally, the small number of participants and the lack of statistical age-matching between the AMD and Control Groups may limit the generalizability of the findings. All said, future work will be focused on recruiting larger, age-matched cohorts to reduce possible confounding effects.

## 5. Conclusions

This study demonstrated that individuals with AMD adopt oculomotor strategies to compensate for central vision loss during real-world reading. Notably, the number of saccades per paragraph was correlated with reading indices, indicating a possible association with reading difficulty in individuals with bilateral central scotoma. While these findings are promising, further replication and longitudinal studies are needed to confirm our results. A key strength of the proposed approach is its multimodal design, which integrates functional assessments of reading indices, microperimetry, structural macular imaging, and eye-tracking analysis. This comprehensive approach enables the investigation of how individuals with AMD adapt to bilateral central scotoma when reading in a real-world setting.

## Figures and Tables

**Table 1:** Calculated parameters to compare the AMD and Control Group with the eye tracking approach (reading duration and 5 parameters calculated from delineated saccades).

Parameter	Explanation	Expectation	Reference(s)
Reading duration [s]	Total time a subject spent reading a specific paragraph of text	Reading duration may be prolonged	[41]
Saccade amplitude [°]	Angle of eye movement from start to end of a saccade	Smaller amplitude varies, usually higher variability	[19,36,42]
Peak saccade velocity [°/s]	Maximum speed during a saccade, around 300°/s for healthy adults	Often reduced or erratic	[36,43,44]
Saccade duration [ms]	Duration from onset to offset of a saccade, typically between 30 and 80 ms	Duration may be prolonged	[36,43]
Number of detected saccades per paragraph	Count of saccadic eye movements detected during reading paragraphs	Increased number of saccades	[18]
Fixation duration [ms]	Duration spent on a single fixation point, approximately 200–300 ms in healthy individuals	Expected to be longer, due impairment in fixation stability	[12]

**Table 2:** List of parameters used for the analysis.

Group	Name of the Parameter
Group 1	age, reading VA, reading speed, near contrast sensitivity, reading duration, saccade amplitude, peak saccade velocity, saccade duration, number of saccades per paragraph, fixation duration
Group 2	ETDRS (LogMAR), static fixation characteristics (circle 2%, circle 4%, static BCEA 1 sd, static BCEA 2 sd, and static BCEA 3 sd)
Group 3	dynamic fixation characteristics (dynamic circle 2%, dynamic circle 4%, dynamic BCEA 1 sd, dynamic BCEA 2 sd, dynamic BCEA 3 sd), PRL-fovea distance, and AMD lesion size

ETDRS—early treatment diabetic retinopathy study, logMAR—logarithm of the minimum angle of resolution, BCEA—bivariate contour ellipse area, PRL—preferential retinal locus, MP—microperimetry.

**Table 3:** Reading indices and static fixation stability data comparing parameters of the better-seeing eye in the AMD Group and parameters of right eye in the Control Group.

Parameter	Descriptive Statistics									
	AMD Group (*N* = 17)					Control Group (*N* = 17)				
	Mean	SD	25%	Median	75%	Mean	SD	25%	Median	75%
Reading VA [logMAR]	0.95	0.22	0.80	0.90	1.10	0.18	0.20	0.00	0.20	0.30
Reading speed [wpm]	37.12	21.95	19.00	38.00	55.00	142.65	42.79	120.00	133.00	150.00
Contrast sensitivity [logCS]	1.03	0.27	0.90	1.05	1.20	1.65	0.14	1.65	1.65	1.80
circle 2 [%]	66.47	32.68	32.00	61.00	100.00	99.24	1.52	99.00	100.00	100.00
circle 4 [%]	89.71	13.92	81.00	95.00	100.00	99.59	0.87	100.00	100.00	100.00
BCEA 1 sd [area]	4.45	5.14	0.30	2.50	7.80	0.35	0.39	0.10	0.02	0.50
BCEA 2 sd [area]	11.98	13.84	0.90	6.80	20.90	0.91	1.03	0.30	0.40	1.30
BCEA 3 sd [area]	22.92	26.49	1.70	13.00	40.00	1.76	1.95	0.60	0.80	2.50

*N*—number of subjects, SD—standard deviation, BCEA—bivariate contour ellipse area, logCS—logMAR—logarithm of the minimum angle of resolution, logCS—logarithm of contrast sensitivity, wpm—words per minute.

**Table 4:** The statistical analysis of eye-specific parameters (Group 2). Statistically significant changes (*p*-value < 0.05) are marked in bold.

Name of the Parameter	KW *p*-Value	Pairwise Comparison	Post Hoc Test *p*-Value	Effect Size	Effect Size Interpretation
ETDRS	<0.001	**CE vs. BE**	**<0.001**	**1.000**	Large
		**CE vs. WE**	**<0.001**	**1.000**	
		**BE vs. WE**	**0.012**	**0.751**	
LogMAR		**CE vs. BE**	**<0.001**	**−1.000**	
		**CE vs. WE**	**<0.001**	**−1.000**	
		**BE vs. WE**	**0.012**	**−0.751**	
circle 2		**CE vs. BE**	**0.007**	**0.574**	
		**CE vs. WE**	**<0.001**	**0.924**	
		**BE vs. WE**	**0.048**	**0.405**	Medium
circle 4		**CE vs. BE**	**0.022**	**0.481**	Large
		**CE vs. WE**	**<0.001**	**0.896**	
		**BE vs. WE**	**0.023**	**0.450**	Medium
BCEA 1 sd		**CE vs. BE**	**0.003**	**−0.678**	Large
		**CE vs. WE**	**<0.001**	**−0.945**	
		BE vs. WE	0.056	−0.439	Medium
BCEA 2 sd		**CE vs. BE**	**0.002**	**−0.709**	Large
		**CE vs. WE**	**<0.001**	**−0.952**	
		BE vs. WE	0.058	−0.446	Medium
BCEA 3 sd		**CE vs. BE**	**0.002**	**−0.709**	Large
		**CE vs. WE**	**<0.001**	**−0.952**	
		BE vs. WE	0.058	−0.446	Medium

ETDRS—early treatment diabetic retinopathy study, logMAR—logarithm of the minimum angle of resolution, KW—Kruskal-Walis, CE—control (right) eye, BE—better-seeing eye of the AMD Group, WE—worse-seeing eye of the AMD Group.

**Table 5:** The descriptive statistics from the better-seeing eye in the AMD group.

Parameter	Descriptive Statistics (*N* = 17)				
	Mean	SD	25%	Median	75%
Dynamical fixation, circle 2 [%]	34.76	24.86	15.00	31.00	44.00
Dynamical fixation, circle 4 [%]	65.12	25.09	53.00	75.00	83.00
Dynamical fixation, BCEA 1 sd [area]	17.82	16.23	7.10	10.30	22.40
Dynamical fixation, BCEA 2 sd [area]	47.96	43.69	19.10	27.70	60.30
Dynamical fixation, BCEA 3 sd [area]	91.79	83.62	36.50	53.00	115.50
PRL-fovea distance [mm]	1.69	0.99	1.05	1.70	2.58
AMD lesion size [mm^2^]	12.34	6.07	6.87	11.39	18.02

AMD—age-related macular degeneration, SD—standard deviation, BCEA—bivariate contour ellipse area, PRL—preferential retinal locus.

## Data Availability

Eye tracker data and tabular parameters are available from the first Author on reasonable request, since the data are part of an ongoing study and will be shared subsequently.

## Abbreviations

The following abbreviations are used in this manuscript:

ANOVA	Analysis of variance
AMD	Age-related macular degeneration
BE	Better-seeing eye of the AMD Group
BCEA	Bivariate contour ellipse area
BCVA	Best-corrected visual acuity
CE	Control (right) eye
DAF	Decreased autofluorescence
ETDRS	Early treatment diabetic retinopathy study
FAF	Fundus autofluorescence
FDR	False discovery rate
H	Horizontal
HV	Horizontal and vertical
I-VT	Velocity threshold
KW	Kruskal-Walis
logCS	Logarithm of contrast sensitivity
logMAR	Logarithm of the minimum angle of resolution
MNREAD-SI	Minnesota low vision reading chart in Slovene
MMSE	Mini mental state examination
MP-3	Microperimeter—third generation
ms	Milliseconds
NA	Not available
OCT	Optical coherence tomography
PRL	Preferential retinal locus
SD	Standard deviation
V	Vertical
VA	Visual acuity
WE	Worse-seeing eye of the AMD Group
wpm	Words per minute
°	Degrees of visual angle
°/s	Degrees per second
